# Lab-on-a-Chip Electrochemical Biosensors for Foodborne Pathogen Detection: A Review of Common Standards and Recent Progress

**DOI:** 10.3390/bios13020215

**Published:** 2023-02-01

**Authors:** Or Zolti, Baviththira Suganthan, Ramaraja P. Ramasamy

**Affiliations:** Nano Electrochemistry Laboratory, College of Engineering, University of Georgia, Athens, GA 30602, USA

**Keywords:** foodborne pathogens, food safety, biosensors, electrochemical sensors, standards, lab-on-a-chip, microfluidics

## Abstract

Foodborne pathogens are an important diagnostic target for the food, beverage, and health care industries due to their prevalence and the adverse effects they can cause to public health, food safety, and the economy. The standards that determine whether a given type of food is fit for consumption are set by governments and must be taken into account when designing a new diagnostic tool such as a biosensor platform. In order to meet these stringent detection limits, cost, and reliability standards, recent research has been focused on developing lab-on-a-chip-based approaches for detection devices that use microfluidic channels and platforms. The microfluidics-based devices are designed, developed, and used in different ways to achieve the established common standards for food pathogen testing that enable high throughput, rapid detection, low sample volume, and minimal pretreatment procedures. Combining microfluidic approaches with electrochemical biosensing could offer affordable, portable, and easy to use devices for food pathogen diagnostics. This review presents an analysis of the established common standards and the recent progress made in electrochemical sensors toward the development of future lab-on-a-chip devices that will aid ‘collection-to-detection’ using a single method and platform.

## 1. Introduction

Foodborne pathogens include different infectious biological agents that cause disease and are found in different food or water products. Each year, the United States federal government estimates that there are 128,000 hospitalizations and 3000 deaths due to foodborne illness annually [1]. Foodborne illness outbreak is defined as at least two illnesses caused by the same pathogen and linked to the same infective source [2]. The year 2021 saw 17 reported foodborne illness outbreaks, resulting in 1424 cases of sickness, 379 hospitalizations, and seven deaths [3]. In addition to the health effects, the last published estimated cost of foodborne pathogens was 17.6 billion USD annually [4]. There is a high interest in the early detection of foodborne pathogens that will help minimize the public health and economic burden caused by foodborne illness.

The detection of foodborne pathogens can be carried out using various methods and kits. The food industry conducts periodic testing for microbial contamination at specific control points during manufacturing and delivery as the standard procedure [5,6]. The gold standard for the detection of foodborne pathogens is culture based and includes visual, biochemical, and immunological means before or after enrichment. In this method, a sample is introduced to a nutrient filled medium, incubated, grown, and plated. It is not ideal due to the time-consuming transportation, robustness, is expensive, and requires skilled labor [7,8,9]. It is even more problematic for industries in remote and rural areas that cannot afford the time and monetary cost of such procedures [10,11]. Due to these restraints, alternative detection methods have been developed where the most common are polymerase chain reaction (PCR) and enzyme-linked-immunosorbent assay (ELISA), which detect the pathogen by finding either a specific DNA or RNA sequence or a specific protein. Both PCR and ELISA will detect the pathogen quickly, but in most cases, cannot distinguish between live and dead bacteria in addition to being incompatible with field conditions [9,12]. Another approach is to develop biosensors; these devices combine a biological recognition element (i.e., bacteriophage, antibody, enzyme, protein, etc.) with a transducer that transforms the interaction between the target pathogen and the biorecognition element to an electrical signal [13].

Electrochemical biosensors (ECBS) have an advantage with respect to other biosensors due to their selectivity, sensitivity, and relative simplicity of use. ECBS have several techniques, differentiated according to the electrical signal that is measured. The most common technique for foodborne pathogens is electrochemical impedance spectroscopy (EIS) due to its very high sensitivity but is limited by the alternating current (AC) potentiostat required for signal generation, which reduces its portability and increases the cost with respect to direct current (DC) methods [14,15,16,17,18,19,20]. In contrast, amperometric methods (i.e., cyclic voltammetry, chronoamperometry, linear sweep, etc.) have an advantage with low cost and an equivalent sensitivity to EIS. Their main disadvantages are the need to continuously correct their calibration due to Faraday’s processes, higher sensitivity to mass transfer limitations in the solution, and relative high applied potential [15,21,22,23,24].

Microfluidics is a term describing the movement of fluids in geometrically restricted dimensions in orders of magnitude of 10^−4^ m or smaller and manipulate them at a µL-nL volume. In the field sensors, the initial use of microfluidics was to improve the sensor’s performance and reduce the consumption of reagents. In addition, microfluidic channels enable the integration of separation, mixing, and monitoring within a single device. Advances in micro and nano fabrication techniques have proven to improve the synergy of new electrochemical biosensors and microfluidic designs to achieve better portability, reduce energy consumption, need of sample pretreatment, and better integrability of the systems into existing production lines. The combination of microfluidics and biosensors have been a major vector in the development of new lab-on-a-chip (LOC) or miniaturized total analysis system (µTAS) platforms [21,25,26,27]. The major reasons for the focus on point of care LOC platforms is their ability to minimize the required pretreatment, automate all fluid handling, and the integration of sample preparation to detection on one easy to use device that is portable and does not require any specially trained personal or additional cost due to expensive equipment [28,29].

Foodborne pathogens ECBS have been the subject of many reviews in recent years. Mei et al. [30] focused mostly on carbon nano-materials on the surface of the electrochemical biosensor and their advantages in detecting foodborne pathogens. Villalonga et al. [31] focused on ECBS for food bioprocess monitoring, and Curulli et al. [32] focused on ECBS for food toxins and contaminants. All three reviews mentioned microfluidic channels, but the focus of those review articles was not specific toward ECBS platforms. Other recent reviews have focused more on the integration of microfluidics to ECBS and other biosensors, but have not placed an emphasis on foodborne pathogens [33,34]. Additionally, none of the foodborne pathogen biosensor reviews discussed and analyzed the established diagnostic standards accepted in the food industry or the health impact of foodborne diseases. Hence, it is clear that the need for a review that combines all three subjects is needed. This review starts with an overview of the established standards regarding foodborne pathogens that set the requirements for current and future detection methods. In addition, recent advances in LOC platforms for the detection of different pathogens will be analyzed, and the design and capabilities of both the microfluidic channels and biosensors will be discussed.

## 2. Foodborne Pathogen Statistics and Standards

**Figure 1:**
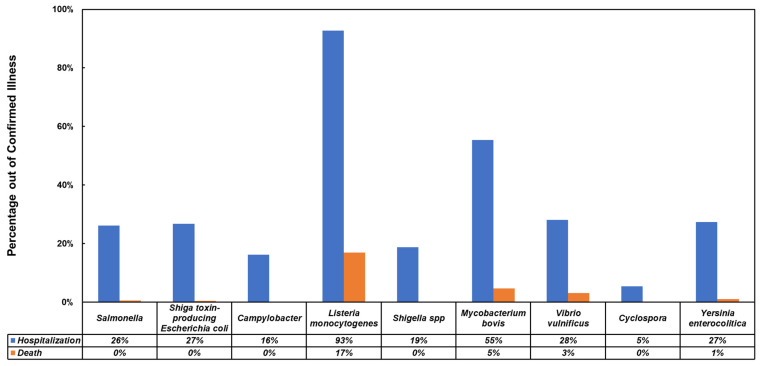
Hospitalization and death rates from confirmed infections with common foodborne pathogens [47,48,49,50].

The standard that governs the standard for the allowed concentration of foodborne pathogens is set by governmental organizations. The standard of whether or not a product is fit for human consumption is set for each specific type of food products separately such as dairy, shellfish, ready to eat foods, etc. While some countries like Great Britain set quantitative limits that correspond to consuming population susceptibility and the infective dose, other countries like the United States of America have mostly set the standard on any detectable trace of these pathogens in the products tested [35,36,37,38,39]. These standards are affected by the minimum infective dose (MID) of each foodborne pathogen, and describe the detectable amount in a specific food or drink. Both the standards, MID, products that contain the pathogen, and the fitness of the tested sample for human consumption for the detected concentration are shown in Table 1. As seen in Table 1, not all pathogens appear in both standards, which is due to its prevalence in that country. These standards will be mostly used during testing prior to the product reaching the consumer or during specific steps in the production and distribution chain [35,36,37,38,39,40]. It is important to also analyze the effect of the most common pathogens on the infected individuals. The biggest issue with such analysis is generated from the nature of the illness, which in most cases will be very mild and will not be diagnosed. The analysis shown in Figure 1 displays the percentage of hospitalization and mortality from the total number of confirmed illnesses in the U.S. between 1996 and 2020. With the exception of Listeria monocytogenes, the mortality rate is low, but the hospitalization rate is over 20% for most of the common pathogens. The hospitalization rates can explain the high annual cost of foodborne pathogens and why they have received the focus as targets for different biosensors [4].

## 3. Microfluidic Channel Material Choice

**Figure 2:**
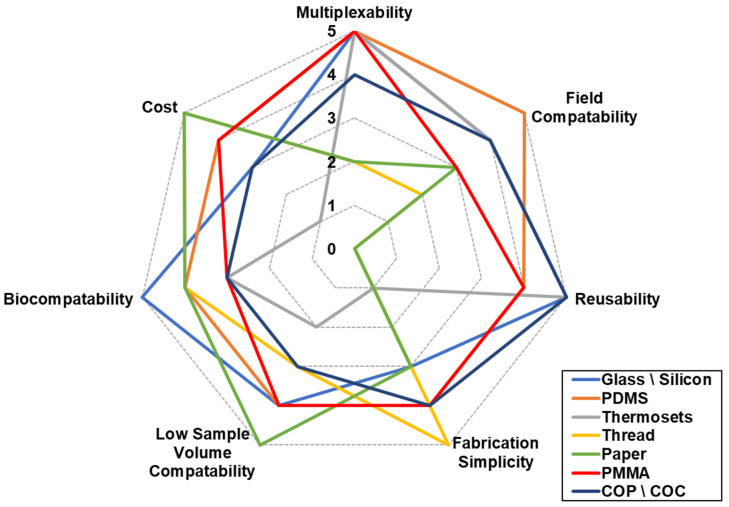
Microfluidic channel material choice according to the main characteristics.

The characteristics of a microfluidic channel are derived from the material used to fabricate them. The channel’s biocompatibility, reusability, fabrication simplicity, and cost are among the first characteristics that come to mind when considering a microfluidic channel for electrochemical biosensor use. In addition to the qualities that are important, their ability to be used in the field, the use of low sample volume, and compatibility to be used with multiple different pathogens (multiplexability) will make a huge impact when they are considered as a viable solution for detection in industrial settings [51,52,53,54]. Focus on microfluidics for foodborne pathogen electrochemical detection since 2017 has shown that the main materials used are glass [55], polydimethylsiloxane (PDMS) [16,17,19,20,56], thermosets [18], paper [57], and thread-based [58]. In addition, there are also other materials that should be considered as microfluidic channel fabrication materials, although not as common such as polymethylmethacrylate (PMMA), cycloolefin polymer (COP), cyclic olefin copolymer (COC), and silicon. These characteristics were evaluated on a scale of 1–5 and are presented in Figure 2 as per channel material, while the details leading to the evaluation are presented below. Some microfluidic channels can be made by the combination of two or more materials and their characteristics will change accordingly. Figure 2 further explains the popularity of glass and PDMS since both display preferable traits in most categories.

### 3.1. Glass

The word glass is used to describe various materials among them: borosilicate [51,59,60], Pyrex [61], soda lime [62], quartz [63], and others [64,65]. Glass microfluidic channels are usually fabricated by using photolithography to print complex patterns on them and etch specific areas to form channels of specific height and width. Due to its amorphic structure, glass etching usually results in a round cross-sectional profile, which can help create a more homogenous flow pattern but forms a challenge when a high aspect-ratio is required [51,59,61]. Other fabrication methods include micromachining, where material is removed from the substrate and bonding or adhering it to another substrate [60] or laser patterning, where a beam of high-energy laser is used to pattern the channel [62,63]. These fabrication methods are highly accurate, where their resolution is determined by the etching chemicals or the resolution of the lithography and laser machines. The machinery usually comes with a very high-price tag and the use of harsh chemicals increases the complexity of the fabrication process. Glass is also thermoconductive, which means that the temperature of the work environment will be limited to a range that will not affect either the sample or the sensor. Another limitation comes from the hardness of glass and its brittle nature, which makes it harder to use in field conditions and makes the addition of valves or bonding very challenging. Although glass is very biocompatible, it is not permeable to gases, which limits the time a live pathogenic sample can survive in it. Additionally, glass has some very attractive qualities. Its resistance to most organic solvents significantly improves its ability to be washed and reused for multiple experiments, and it is very compatible with metal deposition, high surface stability, and as a substrate, it is commonly found, which reduces the overall cost of the devices. Another quality of glass is its electro isolation property, which allows for the incorporation of electrophoresis within it [51,52,53,54,55,66].

### 3.2. Silicon

Silicon microfluidic channel fabrication is very similar to glass. Si microfluidic channels are fabricated by means of micromachining and photolithography and wet etch similarly to the fabrication of glass microfluidics. Another technique that is mostly used in Si is the buried channel technique, where a deep vertical trench is etched into the Si by deep reactive ion etching (DRIE), followed by isotropic etching of the bottom [67]. Another advantage of Si lies in its ability to fabricate thin membranes that can be used to form integrated micropumps [68] and microvalves [69] in the channel. Silicon and glass have very similar characteristics, but one major difference is due to the crystalline structure of silicon, which causes a rectangular cross-sectional shape, while glass has a round one. Another difference between the two is the fact that Si is opaque and will not let light pass through it [51].

### 3.3. PDMS

PDMS is the most popular material used for microfluidic fabrication, in general, and as a microfluidic channel for foodborne pathogen microfluidic electrochemical detection specifically [16,17,19,20,56]. PDMS microfluidic channels are fabricated using a mold, also known as soft lithography, along with low temperature curing, which makes it very repeatable and highly cost efficient. The advantages of PDMS includes the ease of bonding with other PDMS components to form complexed multi-level channels, and hard substrates such as glass or Si to provide mechanical stability. It is very compatible with a high concentration of valves, it is biocompatible, possesses very low toxicity, has a high permeability to gases, which allows a long biostability time of living pathogens in the channel, and supports a very low resolution and any cross-sectional profile, which depends on the mold used. While these advantages make it a very popular material to be used, it also possesses some significant disadvantages. PDMS is hydrophobic and tends to adsorb or absorb small hydrophobic molecules into its walls and cause swelling. It is very sensitive to most organic solvents, which restricts it to aqueous samples only and reduces its reusability. The rigid surface of PDMS can cause pathogens to be trapped in its surface, and its high permeability to gases can change the concentration of a sample due to water evaporation through its walls [51,52,53,54,66].

### 3.4. PMMA

PMMA is a transparent and rigid thermoplastic polymer, which makes it ideal for sustainable applications. It also possesses a glass-like quality with its clarity, UV resistance, low-toxicity, and transparency, with half the density and an order of magnitude better impact resistance. PMMA is chemical resistant and is not affected by aqueous solutions, detergents, inorganic acids, alkalis, and aliphatic hydrocarbons. Among its disadvantages are low impact resistance with respect to other polymers, very limited heat resistance, sensitive to some organic solvents, poor wearing resistance, and tends to crack under medium to high load [70,71]. One of the common fabrication methods is hot embossing, where a piece of PMMA is placed on a Si or metal negative master pattern. The system is then heated under continuous pressure [72]. Another technique is room-temperature imprinting, where the PMMA is placed on a silicon template and then pressed together under high pressure [73]. As with most polymers, PMMA microfluidic channels can also be made with injection molding, where PMMA pallets are melted and injected on a master template under high pressure and then cooled down to room temperature [74]. Finally, as in glass and silicon, PMMA can be molded into microfluidic channels by laser ablation or wet etching [75].

### 3.5. COP/COC

COP and COC are promising materials for microfluidic channels due to their chemical resistance for polar solvents, low water absorption, transparency, ease of fabrication, and bio-inertness [76,77]. COP and COC fabrication methods are laser ablation, injection molding, hot embossing, and nanoimprint lithography. They are also thermoplastic polymers like PMMA and possess a very high electric insulating capability. Their major disadvantage is their low chemical resistance to organic solvents [78,79].

### 3.6. Thermosets

Thermosets such as VisiJet^®^ M2R-CL [18] are very limited in their microfluidic applications, mostly due to their high cost and high stiffness. Their advantages lie with their high chemical and thermal stability, which improves their field compatibility and reusability. Furthermore, their compatibility with 3D printing allows them to be shaped into highly complexed channels, but reduces their ability to work with a low sample volume. Thermosets can also support very high aspect ratio due to their high strength [51,52].

### 3.7. Paper

The fabrication of paper-based microfluidics is conducted by creating hydrophobic barriers on selected areas on the paper substrate to force the sample to flow in a specific path. Paper-based microfluidics are very cost effective. Paper-based microfluidics are also very simple to make, have high porosity and physical absorption, which makes them more compatible with the field. They are easily sterilized and modified, which assists in letting only specific biocomponents through, are biocompatible, and do not require a pump or any other supporting equipment. Alternatively, paper-based systems have very low mechanical properties, and in a more complex design, the sample flow might experience some challenges [54,80].

### 3.8. Thread Based

Thread-based materials for microfluidics have some great advantages with respect to paper-based ones. Thread-based microfluidics are very cost efficient since they do not require a clean room, complex fabrication methods, or expensive machinery. They are therefore also very simple to fabricate, while their hydrophilic and capillary nature make pumps and hydrophobic barriers redundant. Most threads used for bio detection are very biocompatible, easily modified with different biorecognition elements, and can be easily shaped to almost any planar or 3D structure. Their low weight and handling simplicity make them relatively suitable for field work. Additionally, threads cannot be reused, and while they do have high strength compared to paper, overall, they are very sensitive to mechanical strain. Thread-based microfluidics are still only available in laboratory settings and have not yet been used for any commercial applications [58,81,82].

## 4. Microfluidics for Sample Preparation in Electrochemical Biosensors

**Figure 3:**
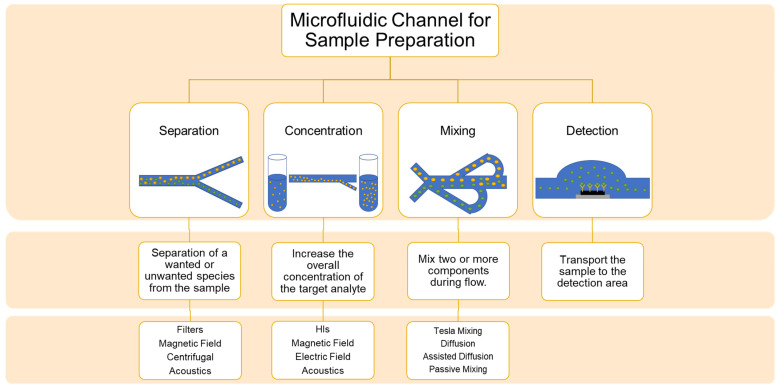
A schematic of the different microfluidic approaches for sample preparation.

Microfluidics for the use of electrochemical pathogenic biosensors can be divided according to their goal in the system [27,60,83]. The goals from recent publications were mapped and found to be focused on separation, concentration, detection, and mixing of reagents before detection, as seen in Figure 3. Examples for each category since 2018 are shown in Table 2, where each example includes a summary of the method of which the goal is achieved, along with the target pathogen, flow characteristics, channel material, and the electrochemical detection technique. A few trends that are emerging from the data are that the majority of microfluidic channels are fabricated with traditional photolithography methods to create a mold out of SU-8 photoresist and use it to create an inversed PDMS channel [16,17,19,20,56,84]; other fabrication methods are micromachining glass [55], cotton thread [58], and 3D printing of polyacrylate [18]. PDMS channels are used since the technology is very established and allows one to form complex 2D designs quickly and accurately when the smallest segment’s dimension is determined by the resolution of the photolithography mask aligner and the type of photoresist used to form the mold. The advantage of 3D printed channels is the ability to create complex 3D shapes without the need for an alignment process. It is also very clear that the majority of microfluidic integrated electrochemical biosensors for foodborne bacteria use EIS as their electrochemical technique [16,17,18,19,20]; differential pulse voltammetry (DPV) was used for the detection of *Norovirus* [56], potentiometric electromotive force (EMF) was used to detect *Salmonella typhimurium* [57], and amperometric techniques were used to detect *Vibrio parahaemolyticus* [58]. It should be noted that most microfluidic channels reported in the literature are meant for multifunctional use, which include two or more of the following functions: separation or isolation, concentration, enrichment, mixing, detection, etc. [83,85].

### 4.1. Microfluidic Separation Channel

Microfluidic separation can be achieved with different methods. The most common approach is the use of microbeads. These microbeads can act as a filter according to their size and concentration within a specific area in the channel [56]. Another use of microbeads is by creating complexes of the target pathogen and magnetic microbeads and separating them by exposing it to a magnetic field [16,86,87]. Except for nanostructures, another separation approach is to utilize mechanical forces such as centrifugal forces to separate the pathogen from the sample. The channels are designed to separate the target pathogen from the sample according to their size or mass [88,89,90]. In addition, another approach for separation is using external forces such as acoustophoretic separation that can separate large particles (>10 µm) from the target pathogens (≤4 µm) by applying ultrasonic acoustic waves [91]. Electrokinetic separation (electrophoretic and dielectrophoretic), which is quite popular for bacterial separation, has not been reported extensively for foodborne pathogens in recent years [92,93].

### 4.2. Microfluidic Concentration Channel

The use of microfluidic channels for concentration and enrichment has been of recent interest due to the small volume most sequencing techniques require (~100 µL for PCR) and the low MID of most foodborne pathogens. Using a concentration microfluidic channel reduces the need for pretreatment processes for the tested sample and lowers the required time from sample collection to detection. One approach to increase the concentration of the pathogens, specifically viruses and bacteria, is the use of homobifunctional imidoesters (HIs) that include positively charged chemical solutions followed by isothermal solid-phase nucleic acid amplification to detect pathogens according to their nucleic acids [94], however, although this method will allow significant amplification of the nucleic acids in the sample, it still requires about an hour of pretreatment and laboratory settings for it to work. Another approach is the use of auxiliary forces such as acoustic waves, magnetic fields, or electric fields. Similar to microfluidic separation, the auxiliary forces are used to remove the target pathogen from the main sample volume and force it into a specific area where the same amount of cells are now in a smaller volume of carrier fluid, which in turn effectively increases the concentration of the tested sample [17,89,90]. In a similar fashion, the use of mechanical forces as described for separation channels, again forces the pathogens into a smaller volume and hence increases their concentration [88,89,90]. The major advantages of using auxiliary forces or internal forces are their time saving and compatibility with the field conditions. It is very clear that when using auxiliary or internal mechanical forces, the channel separates the pathogen and increases its concentration all at once.

### 4.3. Microfluidic Detection Channel

Microfluidic detection channels show the simplest design and are used mostly as a way to bring the sample to the biosensor with the correct flow characteristics, which will allow for the successful detection of the target pathogen. The detection channel will mostly use very low flow rates to avoid kinetic interference from the movement of the particles. They will also be combined with a screen-printed electrode (SPE) platform or a fabricated integrated circuit (IC) to carry out the electrochemical detection [18,19,20,55,84]. A big advantage of these platforms is in their simplicity, which makes their fabrication easily repeatable. Detection channels will also only require a pump of sorts to inject the sample, which will significantly reduce the cost and increase their field compatibility [84].

### 4.4. Microfluidic Mixing Channel

Microfluidic channels pose the biggest design challenge due to the laminar nature of microfluidic flow and its typically very low Reynolds number, which does not allow for the formation of turbulence. Microfluidic mixing channels in the field of biosensing are used mostly to form complexes of the target pathogens with other micro-particles. These complexes will in turn help the specificity and sensitivity of the detection [16,17]. One approach to allow the mixing within a microfluidic channel is a mechanical one, called the Tesla mixing structure. This design has two inlets that are opposite to one another (top of a “T”) and a single outlet that is perpendicular to both (Tail of a “T”) to form a “T” like shape. To further improve the mixing, the tail is designed so that the flowlines will overlaps by creating a back flow at different regions [17,95]. Another approach is a diffusive one; adding a perpendicular channel with a very low flow rate to the main channel will cause the main flow to carry the secondary flow and mix the reagents or assisted diffusion by placing a magnetic stirrer to a specific area in the channel. In addition, a passive approach for mixing can be achieved by adding different barriers, and holes at strategic locations in the channel will also change the flow profile and force mixing of the different reagents, or storing dry and wet reagents at different locations in the channel to be carried by the main flow when the sample is injected [95]. There are also many other microfluidic mixing techniques including electric or magnetic field-based mixing [96,97], ultrasonic or acoustic mixing [98,99], which could be used, but have not been reported in the literature in recent years.

## 5. Lab-on-a-Chip Electrochemical Biosensors

The combination of microfluidics and electrochemical detection on one LOC platform offers the benefits of both worlds such as the small fluid volume and quick processing of microfluidics, along with the sensitivity and specificity of electrochemical biosensors. Electrochemical biosensors can be easily implemented into a microfluidic chip by utilizing modern integrated circuit fabrication techniques [55], microfluidic paper-based technology [57], and 3D printing techniques [18], as seen in Table 2. It is also clear that LOC devices present a relatively quick and accurate detection, as portrayed by the quick detection time of a few minutes [100,101,102,103] and up to no more than 3 h and the LOD of down to 4 CFU/mL for whole bacteria, or 60 copies when the biorecognition element is genome-based [104,105], even when the detection is conducted in complex food matrices, as seen in Table 3. LOC devices also have the ability to combine multiple sensors on one platform to detect multiple pathogens at the same time. This technique, known as multiplexing, utilizes more than one biorecognition element on different detection regions of the device, reduces the consumption of resources, and further reduces the sample pretreatment [55].

LOC portability and field compatibility is an important factor when assessing a device. In addition to the material, the microfluidic channel is made from, as shown in Figure 2, auxiliary equipment such as pumps, readers or a potentiostat, and biorecognition element stability. In most cases, the required auxiliary devices are larger than the LOC device and take up significant space [106], and therefore limits the application for on-field use. The miniaturization of pumps [68,69,107,108] and the use of microcapillaries [109,110] have enabled their integration into the device. In some instances, hand held potentiostats [111,112] integrated with a smart phone reader have been shown to provide better field compatibility [107,109], therefore significantly reducing the need for auxiliary equipment. The stability of the biomolecules of the biosensors presents the biggest challenge for widely used LOC biosensors in the field. Most natural biorecognition elements such as peptides, antibodies, bacteriophage, etc. are very sensitive to environmental conditions. To overcome their stability, molecularly imprinted polymers (MIPs) have been rising as an alternative. MIPs are artificially prepared materials the show advantages with respect to natural biorecognition elements such as reversable adsorption/release of the target pathogen. MIPs can also be imprinted with different nanomaterials to improve their magnetic, optic, or electric characteristics [113,114,115].

LOC offers not only the combination of microfluidics and detection, but also the automation of the whole process. For example, a device using microfluidics to bring the sample to the electrochemical biosensor that utilizes amperometric tests to detect *Escherichia coli* O157:H7 by using horse radish labeled antibody as the biorecognition element and forms an immobilized Ab/bacteria/anti-E. coli antibody structure following exposure to the bacteria [124]. Another interesting approach is the use of paper science to fabricate LOC devices. Paper offers an easier way to transport the fluid and allows for complex 2D structures to be utilized with the use of a printer and specialized ink. They are very portable and can not only deliver the sample to the sensing area, but can also merge multiple reagents, split samples, and delay the delivery by creating hydrophilic or hydrophobic areas on the paper [104,125]. This technology was shown to create a disposable impedimetric biosensor with immobilized antibodies as its biorecognition element. The paper itself acted as the microfluidic channel and carried the sample to the desired location [57]. On the other hand, paper-based LOC platforms tend to lack in sensitivity and their reproducibility is also a big issue that has yet to be solved [126]. LOC platforms can also utilize auxiliary forces such as dielectrophoresis [17,55] or magnetic field [16] to manipulate, focus, and concentrate the sample. They can do so by adding focused electrodes to form the electric field for dielectrophoresis, while an electric coil or a strong magnet can form the magnetic field. Applying such forces have been reported to enhance signal response by up to 18-fold when compared to the reaction without them [55]. Although these methods offer significant advantages and their fabrication is relatively established and common, they also add significant cost and energy requirements to the device.

## 6. Conclusions

This article began with a review of the impact of foodborne illnesses on public health and the economy. Further in this review, the established standards for foodborne pathogen diagnosis for a range of food matrices and pathogen types were discussed in detail. The types of different foodborne pathogens, along with their minimal infective doses and recommended standards for human consumption, were also discussed. The review comprehensively discusses the various types of microfluidic platforms that have been developed and reported for biosensing applications, with an emphasis on electrochemical-based platforms for foodborne pathogens. The review also discusses the distinction between platforms that can solely focus on detection versus the platforms that combine sample preparation and detection on a single device. The importance of material choices for microfluidic platforms based on the desired sensitivity, selectivity, reusability, portability, and field suitability for end application, was critically reviewed in this article. The use of MIPs instead of the commonly used natural biorecognition elements could significantly improve the shelf life and stability of ECBS platforms. Moreover, the use of hand-held potentiostats, integrated capillaries, micropumps, and valves as part of the LOC instead of using syringes and other large auxiliary equipment will aid in the simplification of these platforms for field use.

Future Directions: The research in the field of microfluidic electrochemical biosensors points to the development of devices that will combine all steps from sample preparation to detection including separation, isolation, enrichment, concentration, mixing, etc. Another trend is to develop LOC with multiplexing capabilities to process large volume samples and to screen multiple pathogens simultaneously. Finally, the integration of LOCs with a smartphone-based user interface could enable the easier adoption of these devices by the food industry for in house testing.

## Figures and Tables

**Table 1:** Common foodborne pathogens, infection sources, epidemiological data, and standards.

Foodborne Pathogen	Food Products [35,41,42]	USDA Minimum Infective Dose (MID) Levels [Cells] [36,41,43,44,45,46]	British Standard [CFU/g] [35,36]	FDA Standard [CFU/g] [37,38,39]	Meaning [35,36,37,38,39]
*Campylobacter* spp.	Poultry, beef, dairy products, and untreated drinking water.	xa400–500	>0.04	Any detectable presence ^c^	Potential health hazard and unfit for human consumption
			<0.04	No detectable presence ^c^	Fit for human consumption
Shiga toxin-producing *Escherichia coli*	Under cooked beef, dairy products made with unpasteurized milk or post pasteurization contaminated milk, vegetables, and untreated drinking water.	10–100	>100	Any detectable presence ^c^	Not fit for human consumption
			20–100	No detectable presence ^c^	Farther testing is needed mostly still fit for human consumption
			<20		Fit for human consumption
*Salmonella typhimurium*	Eggs, poultry, pork, beef, dairy products, seeds, herbs, vegetables, chocolate.	15–20	>0.04	Any detectable presence ^c^	Potential health hazard and unfit for human consumption
			<0.04	No detectable presence ^c^	Fit for human consumption
*Listeria monocytogenes*	Poultry, pork, beef, dairy products, bread, fish	Unknown. May vary with the strain and susceptibility of the individual.	>100	>100 ^d^	Not fit for human consumption
			10–100		Not fit for vulnerable groups consumption (e.g., hospital food)
			<10	<100 ^d^	Fit for human consumption
*Shigella* spp.	Foods that are consumed raw, fruits, vegetables, recreational water, water contaminated with stool.	^a^ 10	^a^ *S. sonnei* < 500 CFU	NA	Potentially injurious to health and/ or unfit for human consumption
			^a^ *S. dysenteriae* <200 CFU		
			^a^ *S. flexneri* < 140 CFU		
			^a^ Virulent strain < 10 CFU		
*Mycobacterium bovis*	Contaminated, unpasteurized dairy products, cattle, bison, elk, and deer. Can be acquired through air or wounds of contaminated animal.	^b^ 1 CFU	NA	NA	Potentially injurious to health and/ or unfit for human consumption
*Vibrio vulnificus*	Raw or undercooked oysters and other seafood	10^6^ (10^2^ in predisposed persons)	NA	>30 [MPN/g] ^e^	Not fit for human consumption
*Yersinia enterocolitica*	Raw or undercooked pork	10^9^	NA	Any detectable presence ^c^	Not fit for human consumption
*Norovirus*	Contaminated food, drinks, surfaces, or people	10–100 viral particles	NA	NA	Potentially injurious to health and/ or unfit for human consumption
*Rotavirus*	Stool particles in food and drinks due to bad hygiene.	10–100 viral particles	NA	NA	Potentially injurious to health and/ or unfit for human consumption
*Cyclospora*	Water, fresh produce, food or water contaminated with stool.	Unknown, predicted to be as low as 200 oocysts.	NA	NA	Potentially injurious to health and/ or unfit for human consumption

^a^ Infective dose (ID_50_) (i.e., the oral dose required to cause disease in 50% of healthy adult volunteers challenged with a virulent strain of the pathogen). ^b^ Minimum infective doses in cattle. ^c^ In dairy products according to section 402(a)(1) of the Act (21 U.S.C. 342(a)(1)). ^d^ In ready to eat foods according to section 402(a)(1) of the Act (21 U.S.C. 342(a)(1)). ^e^ In shellfish according to the 2009 NSSP Guide for the Control of Molluscan Shellfish, FDA.

**Table 2:** The different uses of microfluidic channels for electrochemical biosensors.

Goal	Methodology	Foodborne Pathogen	Flow Rate [µL/min]	Sample Volume [µL]	Concentration Range	Channel Material	Detection Technique	Ref.
Separation	Gradient magnetic field.	*Listeria monocytogenes*	1000	3500	10^2^–10^5^ [CFU/mL]	PDMS	Impedimetric phase shift analysis	[16]
	SiO_2_ microbeads separation.	*Norovirus*	NA	NA	100 [pM]–3.5 [nM]	PDMS	Differential pulse voltammetry	[56]
Concentration	Dielectrophoresis	*Escherichia coli* O157:H7	2 [µL/min]—sample flow4 [µL/min]—wash flow4 [µL/min]—Ag enhancement	20 [µL]—sample8 [µL]—wash8 [µL]—Ag enhancement	10^3^–10^5^ [CFU/mL]	PDMS	Impedimetric	[17]
	Positive dielectrophoresis	*Escherichia coli* O157:H7*Salmonella typhimurium*	1–2 [µL/min]	1000	10–120 [Cells/mL]—*Salmonella typhimurium*13–1000 [Cells/mL]—*Escherichia coli* O157:H7	Glass	Impedimetric	[55]
Detection	Transporting the solutions to the sensor	*Escherichia coli* Crooks Strain	180	100	10^5^–10^8^ [Cells/mL]	Polyacrylate	Impedimetric	[18]
	Transporting the solutions to the sensor	*Listeria monocytogenes*	Sample was dripped into specifically made wells on the microfluidic chip.	80	10^2^–10^3^ [CFU/mL]	PDMS	Impedimetric	[19]
	Transporting the solutions to the sensor	*Salmonella (B and D)*	2	NA	290–1000 [Cells/mL]	PDMS	Impedimetric	[20]
	Polymer coated paper modified with PAMAM(NH2)64-Ab	*Salmonella typhimurium*	NA	5000	10^1^–10^8^ [Cells/mL]	Paper	Potentiometric	[57]
	Cotton thread carried the sample to an aptamer with functionalized MoS_2_ nanosheets	*Vibrio parahaemolyticus*	NA		10^1^–10^6^ [CFU/mL]	Cotton thread	CV and DPV	[58]
Mixing	Tesla mixing structure	*Escherichia coli* O157:H7	2	20	10^3^–10^5^ [CFU/mL]	PDMS	Impedimetric	[17]
	Magnetic stirring	*Listeria monocytogenes*	2000	205	10^2^–10^5^[CFU/mL]	PDMS	Impedimetric Phase shift analysis	[16]

**Table 3:** Different examples for the electrochemical platform for foodborne pathogen detection.

Food Borne Pathogen	Detection Technique	Bio-Recognition Event	Sample Type	Analysis Time	Detection Range/Detection Limit	References
*Listeria monocytogenes*	Amperometric	Antigen-antibody	Milk	NR	10^2^ to 10^6^ [CFU/mL]	[116]
	Impedimetric	Antigen-antibody	Filtered tomato extract	NR	4 [CFU/mL]	[117]
	Impedimetric	Magnetic nanoparticles-antibody-urease	Spiked lettuce	NR	3 × 10^2^ [cells]	[118]
	Impedimetric	Modified magnetic nanoparticles—antibody-urease	Spiked lettuce	1 h	1.6 x 10^2^ [CFU/mL]	[16]
	Impedimetric	Micro-electrodes functionalized with antibodies—miniaturized, portable EIS biochip	Milk	NR	55 [CFU/mL]	[19]
	Impedimetric	Immunomagnetic nanoparticles-urease -screen-printed electrode	Spiked lettuce	<3 h	1.6 x 10^3^ [CFU/mL]	[119]
*Campylobacter jejuni*	Amperometric	Antibody, phosphatase	Turkey carcass wash	2.5 h	10^2^–10^7^ [CFU/mL]LOD = 2 × 10^4^ [CFU/mL]	[120]
	Amperometric	Antibody	Milk	<1.5 h	1 × 10^3^–5 × 10^5^ [CFU/ mL]LOD = 4 × 10^2^ [CFU/mL]	[121]
*E. coli* O157:H7	Impedimetric	Antibody	Ground beef	NR	2.05 × 10^3^ [CFU/gr]	[122]
*Salmonella typhimurium*	Amperometric	Antibody	Milk	125 min	10 [CFU/mL]	[100]
	Impedimetric	Aptamer	Apple juice	45 min	NR	[101]
	Impedimetric	Aptamer—Diazonium base	Apple juice	30 min	NR	[102]
	Impedimetric	Interdigitated electrode array coated with Salmonella antibody	Ready to eat turkey	1 h	300 cells/mL	[20]
*Staphylococcus aureus*	Impedimetric	Antibody	Spiked milk	~30 min	13 [CFU/mL]	[103]
	Potentiometric	Aptamers	Pig skin	NR	2.4 × 10^3^–2.0 × 10^4^ [CFU/mL]	[123]
*Norovirus*	Cyclic voltammetry	Selective capture agent concanavalin A	Lettuce extract		60 copies/mL	[104]
	Immuno-based electrochemical biosensor	Monoclonal antibody	Clinical fecal sample	1 h	10^4^ copies/mL	[105]

## Data Availability

The data presented in this study are openly available in the FDA website at reference number [1], CDC website at reference number [3], and FoodNet surveillance reports at reference number [50].
